# The burden of performing minimal access surgery: ergonomics survey results from 462 surgeons across Germany, the UK and the USA

**DOI:** 10.1007/s11701-021-01358-6

**Published:** 2022-02-02

**Authors:** Jonathan Morton, Grant D. Stewart

**Affiliations:** 1 Department of Colorectal Surgery, Addenbrookes Hospital, Cambridge University Hospitals NHS Foundation Trust, Cambridge, UK; 2 Department of Surgery, University of Cambridge, Cambridge, UK

**Keywords:** Minimal access surgery, Laparoscopy, Ergonomics, Musculoskeletal, Survey, Early retirement

## Abstract

This international study aimed to understand, from the perspective of surgeons, their experience of performing minimal access surgery (MAS), to explore causes of discomfort while operating and the impact of poor ergonomics on surgeon welfare and career longevity across different specialties and techniques. A quantitative online survey was conducted in Germany, the UK and the USA from March to April 2019. The survey comprised 17 questions across four categories: demographics, intraoperative discomfort, effects on performance and anticipated consequences. In total, 462 surgeons completed the survey. Overall, 402 (87.0%) surgeons reported experiencing discomfort while operating at least ‘sometimes’. The peak professional performance age was perceived to be 45–49 years by 30.7% of surgeons, 50–54 by 26.4% and older than 55 by 10.1%. 86 (18.6%) surgeons felt it likely they would consider early retirement, of whom 83 were experiencing discomfort. Our findings highlight the continued unmet needs of surgeons performing MAS, with the overwhelming majority experiencing discomfort, frequently in the back, neck and shoulders, and many likely to consider early retirement consequently. Innovative solutions are needed to alleviate this physical burden and, in turn, prevent economic and societal impacts on healthcare systems resulting from MAS limiting surgeon longevity.

**Supplementary Information:**

The online version contains supplementary material available at 10.1007/s11701-021-01358-6.

## Introduction

Minimal access surgery (MAS) has continually improved over recent decades through technological advances in surgical instruments, visualisation and refinement of procedures [1–4]. Consequently, MAS is established as a routine approach for an increasing range of surgeries including gynaecological, colorectal and urological procedures [5, 6]. Compared with open surgery, MAS is associated with less intraoperative blood loss, fewer wound complications, and reduced blood transfusion rates and length of hospital stay [6–8]. These advantages are largely owing to the small size of the incision required at the surgical site. However, laparoscopic instruments required for performing MAS present technical challenges for surgeons despite advances in their designs [4], including limited dexterity and the inversion and scaling of movements, known as the ‘fulcrum effect’ [9]. Consequently, the ergonomics of performing MAS procedures, i.e. the measurement of surgeons’ muscular effort, movements and positioning of the body, is a focus of MAS research [10–12]. Studies assessing surgeon ergonomics while performing MAS demonstrate awkward positioning with prolonged static postures including the following: extended periods of neck rotation, asymmetrical loading between shoulders and frequent adoption of extreme shoulder positions [12–14]. Poor ergonomics can cause muscle fatigue and musculoskeletal injuries, which may have economic implications and limit patient access to surgery due to work absence and potentially early retirement [13]. A systematic literature review (SLR) and meta-analysis of surgical ergonomics reported that generalised pain and fatigue were experienced by 68% and 71% of more than 5000 surgeons, respectively [15].

To date, most studies examining ergonomics in MAS have focused on the direct impact of poor ergonomics, e.g., pain and fatigue. Few studies have directly compared the surgical techniques and specialties or examined the long-term ramifications, such as the impact on a surgeon’s career. This study aimed to understand, from the perspective of a large number of surgeons from multiple countries across different specialties and techniques, their experience of performing MAS. The survey also aimed to explore the causes of MAS-related discomfort and its impact on surgeon welfare and career longevity.

## Materials and methods

### Study design

A quantitative online survey was conducted in three countries: Germany, the UK and the USA. Surgeons completed the survey between 11 March and 2 April 2019.

### Surgeon inclusion criteria and screening process

Surgeons were identified for recruitment from online physician panels, which may be joined through colleague referral, online media or recruiter invitation. Surgeons who had signed up to participate in market research when joining the panels were selected for email invitation via random sampling of those coded as surgeons. To qualify for participation, surgeons had to: be in Germany, the UK or the USA; specialise in colorectal or gynaecological surgery, or be general surgeons performing hernia repair; have practiced as a surgeon for at least two years; routinely conduct at least one of open surgery, MAS and robot-assisted surgery (RAS).

### Ergonomics survey

The online survey comprised 17 questions across four categories: demographics, intraoperative discomfort, effects on performance and anticipated consequences (Supplementary Table S1). Seven questions related to demographics: gender, age, number of years practicing as a surgeon, surgical specialty, height and surgical glove size (extra-small [XS; size 5.5], small [S; size 6.0–6.5], medium [M; size 7.0–7.5], large [L; size 8.0–8.5] or extra-large [XL; size ≥ 9.0]). Ten questions related to frequency and location of physical discomfort when performing MAS procedures; healthcare professional consultation due to MAS-related musculoskeletal injuries; perceived peak surgical performance and likelihood of early retirement due to the physical impact of performing MAS.

### Statistical analyses

Surgeon demographic data were reported using means and SD, and statistical significance (α = 0.05) was determined by Chi-squared test for categorical variables and one-way ANOVA for continuous variables. Statistical analyses were performed using R, version 4.0.0. Statistical analyses were not performed on the survey outcomes data as this was a market research study.

## Results

### Surgeon demographics

In total, 462 surgeons qualified and completed the survey. There was no difference in the representation between surgical specialties across the three countries (*p* = 0.147). Most participants were male (77.1%), mean age was 48.6 (SD: 9.7) years and the range of years practicing as a surgeon was 2–40 (median: 19; Table 1). Most surgeons were regularly performing open surgery (95.5%) and MAS procedures (96.8%), and 33.8% were regularly performing RAS.

**Table 1:** Surgeon demographics

Demographic	UK (*n* = 152)	USA (*n* = 158)	Germany (*n* = 152)	Total (*N* = 462)
Male, n (%)	123 (80.9)	111 (70.3)	122 (80.3)	356 (77.1)
Age (years), mean (± SD)	47.7 (9.4)	48.1 (11.1)	49.9 (8.1)	48.6 (9.7)
Years practising as a surgeon, *n* (%)				
< 5	4 (2.6)	12 (7.6)	1 (0.7)	17 (3.7)
5–10	20 (13.2)	37 (23.4)	9 (5.9)	66 (14.3)
11–20	61 (40.1)	59 (37.3)	74 (48.7)	194 (42.0)
21–30	55 (36.2)	40 (25.3)	58 (38.2)	153 (33.1)
> 30	12 (7.9)	10 (6.3)	10 (6.6)	32 (6.9)
Surgical procedures performed, *n* (%)				
Gynaecology	71 (46.7)	84 (53.2)	80 (52.6)	235 (11.0)
Colorectal	68 (44.7)	62 (39.2)	70 (46.1)	200 (43.3)
General (hernia repair)	37 (24.3)	61 (38.6)	41 (27.0)	139 (30.1)
Height, *n* (%)*				
< 160	5 (3.3)	11 (7.0)	2 (1.3)	18 (3.9)
160–170	46 (30.3)	49 (31.0)	24 (15.8)	119 (25.8)
171–184	76 (50.0)	75 (47.5)	84 (55.3)	235 (50.9)
> 185	20 (13.2)	23 (14.6)	42 (27.6)	89 (19.3)
Glove size, median (range)	7.5 (6.0–8.5)	7.5 (5.5–8.5)	7.5 (5.5–8.5)	7.5 (5.5–8.5)

*Height not recorded for five UK surgeons, *n* = 147

### Discomfort performing surgery

**Fig. 1:**
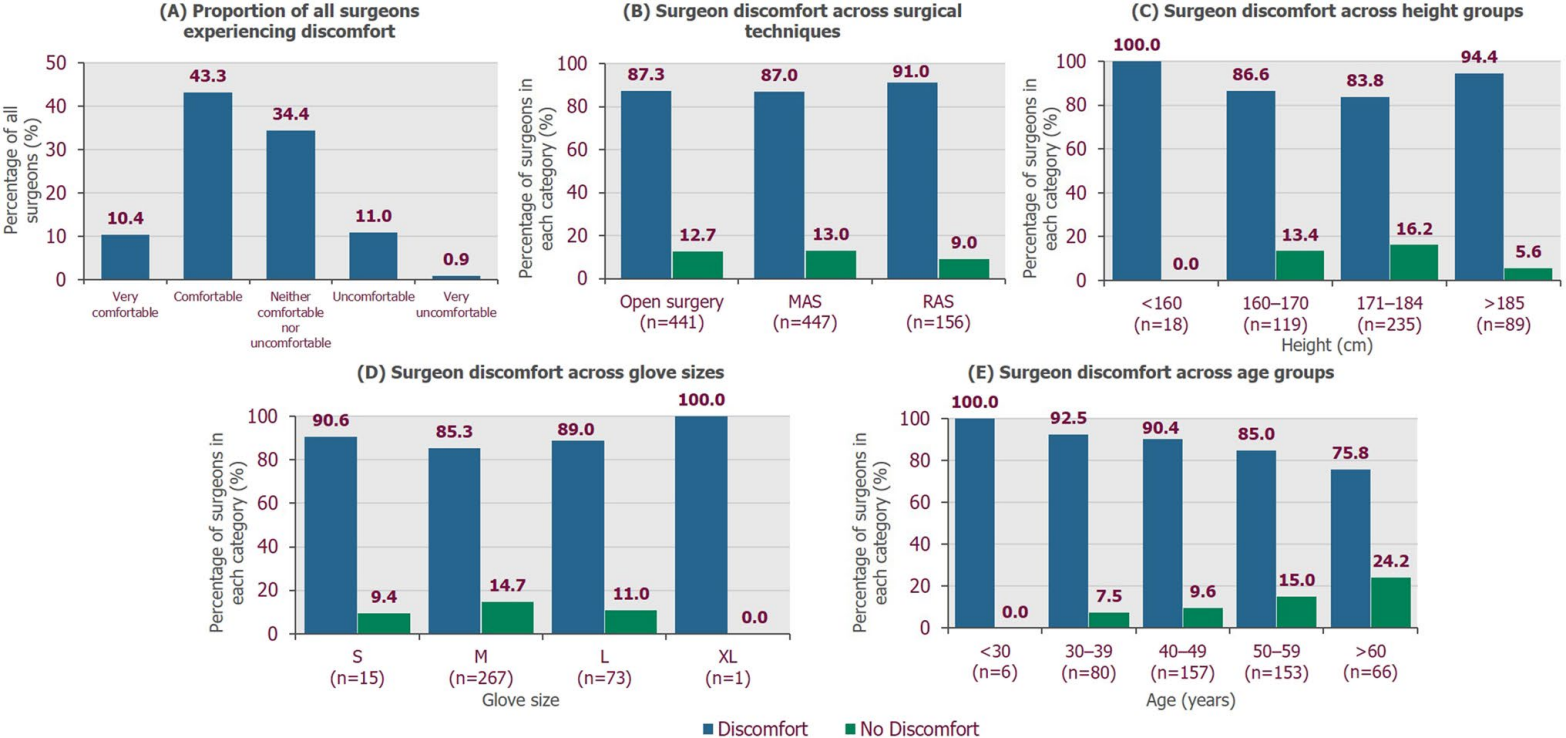
Discomfort while performing surgery. *MAS* minimal access surgery, *RAS* robot-assisted surgery

Overall, 87.0% of surgeons reported experiencing discomfort at least ‘sometimes’ while performing surgery. Although 43.3% of surgeons reported they were generally ‘comfortable’, 34.4% were neither ‘comfortable nor uncomfortable’, and 11.9% reported they were ‘uncomfortable’ or ‘very uncomfortable’ when operating (Fig. 1a). When stratified by sex, the proportions of surgeons reporting discomfort were similar between male and female surgeons. In Germany, the UK and the USA, the proportions of surgeons reporting physical discomfort were 61.8% (94/152), 73.7% (112/152) and 75.3% (119/158), respectively. Discomfort levels were similar across surgical techniques; respectively, 87.3% (385/441), 87.0% (389/447) and 91.0% (142/156) of surgeons reported experiencing discomfort at least ‘sometimes’ in open surgery, MAS and RAS (Fig. 1b).

When stratifying by surgeon height, a greater proportion of surgeons experienced discomfort than did not in all height categories (Fig. 1c). More than 90% of surgeons either < 160 cm or > 185 cm reported discomfort, compared to approximately 85% of surgeons in the 160–184 cm range. Additionally, the proportion of surgeons reporting discomfort ‘frequently’ or ‘every time I operate’ was higher in the < 160 cm and > 185 cm height groups (27.8% and 26.9%, respectively), compared with the 160–170 cm and 171–184 cm groups (23.5% and 17.9%, respectively; Supplementary Fig. S1). Similar results were observed when stratifying by surgical glove size; those with glove size S or XL reported discomfort more frequently compared with the size M and L groups (Fig. 1d).

**Fig. 2:**
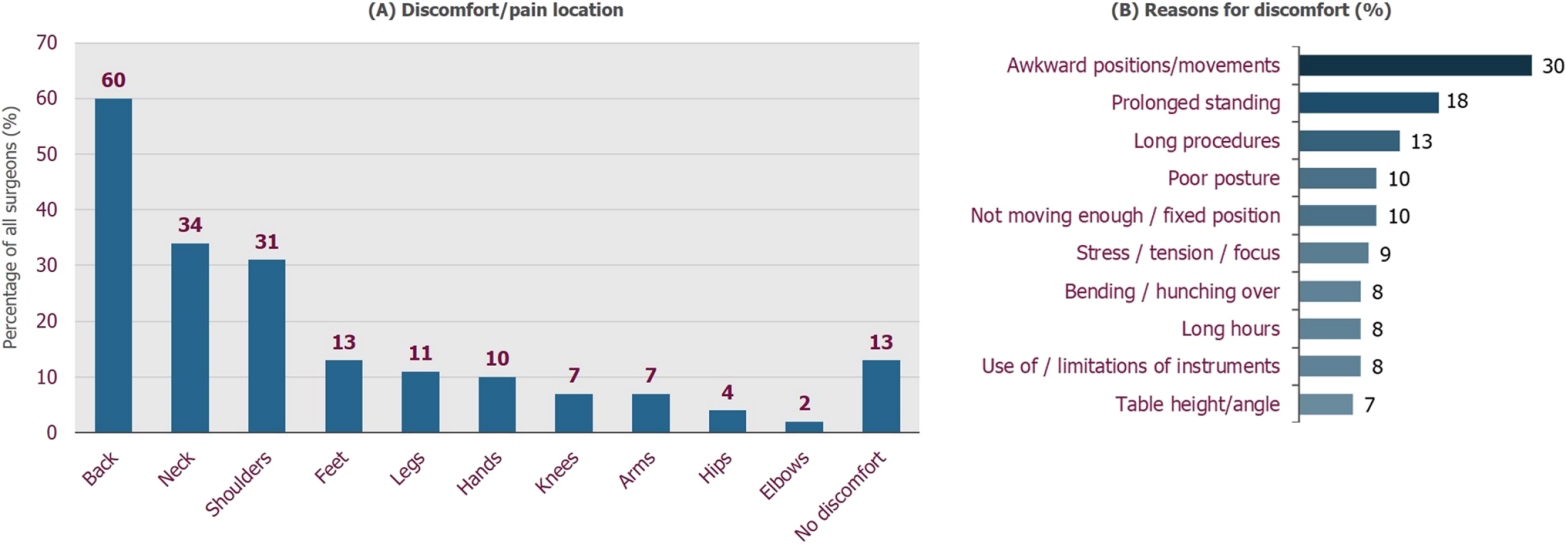
Areas of discomfort and cited reasons for discomfort. **A** Areas of the body most affected by discomfort during surgery (*N* = 462). **B** Percentage of surgeons experiencing discomfort (*n* = 402) who cited categorised reasons for discomfort when performing MAS procedures (direct quotes listed in Supplementary Table S2)

The proportion of surgeons experiencing discomfort was highest in the younger age groups, with all surgeons < 30 years and 92.5% of surgeons aged 30–39 years reporting discomfort (Fig. 1e). Discomfort appeared to affect fewer surgeons in the older age groups; however, in the > 60 years group, which was the least affected, 75.8% (50/66) of surgeons reported discomfort. The area of the body most affected by pain or discomfort was the back, reported by almost two-thirds of all surgeons. More than a third of surgeons reported pain or discomfort in their neck and/or shoulders, and 13.2% reported that their feet were commonly affected when operating (Fig. 2a). Awkward positions/movements, prolonged standing and long procedures were the most cited reasons for discomfort (Fig. 2b). Other causes included poor posture, fixed positions, stress and tension, bending/’hunching over’, long hours, use of/limitations of instruments and table height/angle (Supplementary Table S2).

### Peak performance and early retirement

**Fig. 3:**
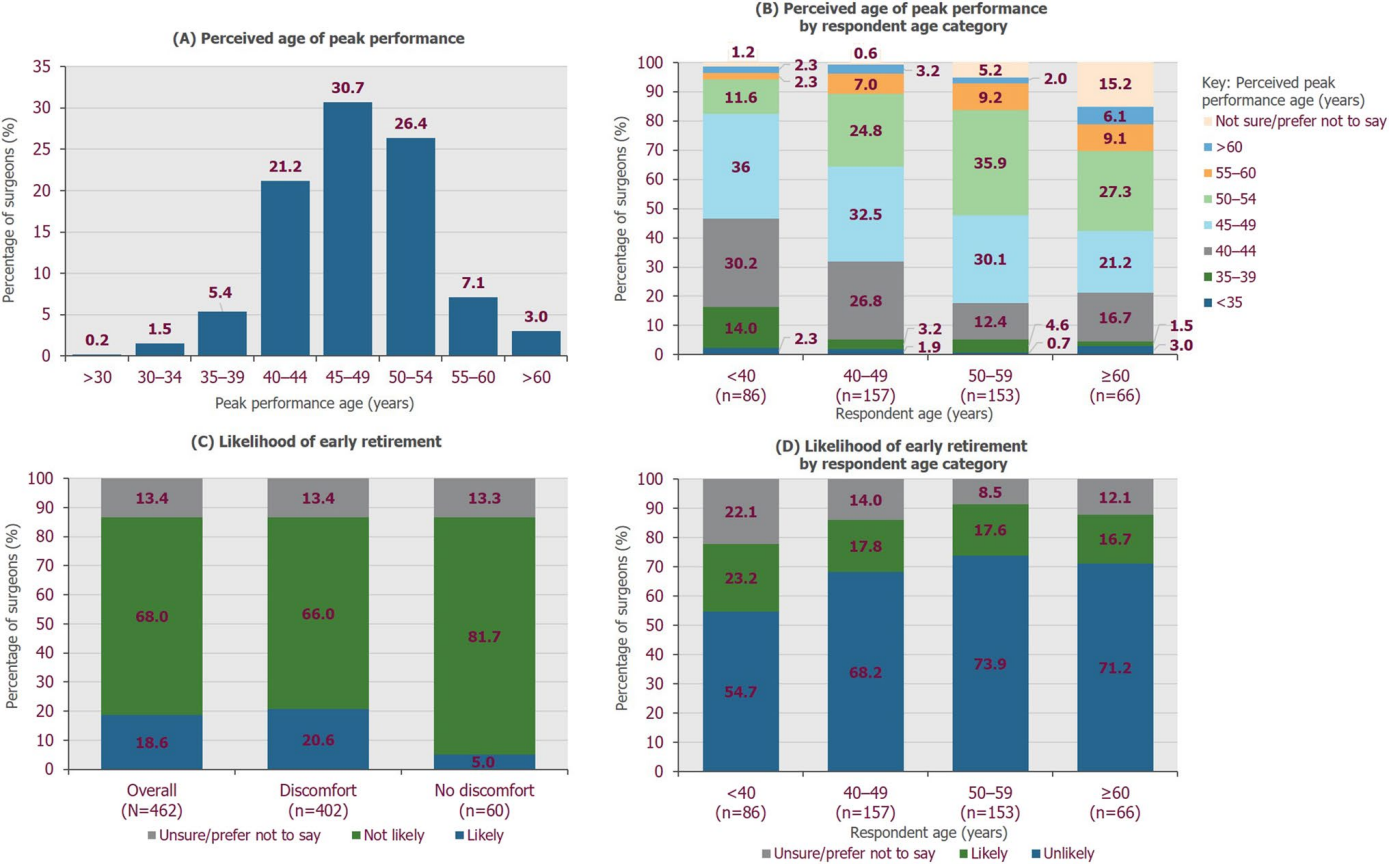
Age of peak performance and likelihood of early retirement. **A** Surgeons’ perceived age of peak surgical performance (*n* = 462). **B** Perceived age of peak surgical performance stratified by respondent age category. **C** Proportion of surgeons experiencing discomfort or no discomfort within each response regarding likelihood of early retirement due to the physical impact of performing MAS procedures. **D** Likelihood of early retirement stratified by respondent age category

The age of peak professional performance was perceived to be 45–49 years by 30.7% of surgeons, 50–54 years by 26.4% and > 55 years by 10.1%; 5.4% and 1.7% of surgeons perceived peak performance age to be 35–39 years and < 35 years, respectively (Fig. 3a). Surgeons tended to perceive peak performance as close to their current age. More surgeons aged 50–59 years perceived peak performance age to be 50–54 years (35.9%; 55/153) compared to those aged < 40 years (11.6%; 10/86; Fig. 3b). Peak performance age was considered to be 40–44 years by 30.2% and 26.8% of surgeons aged < 40 years (26/86) and 40–49 years (42/157), respectively, compared to 13.7% of surgeons aged ≥ 50 years (30/219).

Overall, 18.6% of surgeons felt it likely they would consider early retirement, versus 68.0% who felt it unlikely and 13.4% who reported they were unsure or preferred not to say (Fig. 3c). A greater proportion of surgeons reported they would likely consider early retirement if experiencing discomfort than if not; 20.6% of surgeons experiencing discomfort (83/402) reported it at least ‘fairly likely’ they would retire early, compared with 5.0% of those who reported no discomfort while performing surgery (3/60; Fig. 3c). In the aged < 40 years group, 23.2% of surgeons reported it likely that they would consider early retirement, which was a higher proportion than in the older groups (Fig. 3d).

## Discussion

Most surgeons reported experiencing discomfort at least ‘sometimes’ when performing MAS procedures, with many experiencing discomfort frequently or at every time of operating. Surgeons who were outside of the 160–184 cm height and M/L glove size ranges appeared most affected, suggesting that those at the extremes of these scales may be at higher risk of discomfort. Therefore, surgical systems designed with greater flexibility to suit the individual are required. Our finding that the proportion of surgeons experiencing discomfort was lower in older groups could reflect that the laparoscopic surgery had not yet been fully established in the early decades of their career. It is also possible that the older and likely more experienced surgeons had a shorter average operative time, resulting in less time spent in an uncomfortable position and hence less discomfort. Peak surgical performance age was perceived to be 45–49 years by most, and > 50 years by more than 25% of surgeons. Peak surgical performance has been previously reported at older ages, up to and beyond 60 years old, measured by both in-hospital patient mortality rates [16, 17], and average annual case volume [17]. The tendency for younger surgeons to perceive peak performance age to be younger than average in this study was perhaps to be expected, given that it may be difficult for younger surgeons to predict their future performance. Although retirement age may vary by country, a substantial proportion of surgeons in this study believed they would consider early retirement as a direct result of the physical detriment of performing MAS procedures.

### Ergonomic challenges are inherent in MAS

MAS procedures require surgeons to adopt awkward and static postures for prolonged periods, with angled neck and asymmetrical shoulder positioning and frequently extreme elbow flexions [18]. A case series study, measuring surgeon posture during surgery using wearable technologies, found surgeons spent 65% of procedure time in neck positions classified as ‘high risk’ by occupational ergonomic research exposure–response analyses, and that a surgeon’s cervical spine is at ‘unacceptably’ elevated risk [19]. Similarly, surgeons in our study cited poor body positioning as a cause of neck and back pain when operating, which can be exacerbated in patients with high body mass index (BMI), as the operating table does not adjust far enough for correct positioning (Supplementary Table S2). Surgeons expressed that ‘particularly during laparoscopic procedures, a crooked posture […] leads to long-term muscle tensions and pain in the neck area’, and that ‘especially in laparoscopic procedures, a longer-term oblique posture […] results in neck tension pains’. These findings suggest that improvements in MAS instrument designs have not eliminated surgeon discomfort, and that ergonomic challenges are inherent to the conventional approach to MAS. Although robotic systems can help overcome some ergonomic challenges, issues, such as eye strain and hand/finger stress, have been reported [20].

### Impact on surgeons and socioeconomic consequences

As a consequence of musculoskeletal strains and injuries resulting from poor ergonomics in MAS, surgeons may have to take work absence and many consider early retirement [21]. An SLR reported musculoskeletal pain to be the most common occupational disease in Europe [22]. Additionally, Plerhoples et al. reported that nearly a third of 1215 surgeons admitted to giving consideration to their own discomfort when choosing an operative modality [23]. Our finding that most surgeons perceived the age of peak professional performance in MAS to be later in a surgeon’s career highlights the prolonged learning curve associated with laparoscopic instruments and the extensive experience required for highest-level performance. Considering nearly 20% of surgeons in this study reported they would consider retiring early as a result of the physical impacts of performing MAS procedures, surgeons may not reach their peak professional performance, or reduce the time spent at their peak, before retiring. Consequently, there is a risk of losing a highly experienced surgeon group due to poor ergonomics. In the long term, this could have economic impacts on healthcare systems and could limit patient access to surgery, with growing waiting lists due to lowercase throughput.

### Current approaches towards mitigating the impacts of poor ergonomics in MAS

The findings of this study are not dissimilar to those of a survey study conducted over a decade ago; Park et al. found that 86.9% of laparoscopic surgeons reported physical symptoms or discomfort [24]. As such, solutions to these ergonomic challenges have been needed for some years. Ergonomic guidelines that advise surgeons on arranging their MAS equipment (table height and video feed positioning) to minimise musculoskeletal strain are available [14, 25, 26]. However, studies suggest surgeons may not be aware of these guidelines [11, 12, 24, 27]. One current strategy towards mitigating the mental and physical strain of performing MAS procedures is intraoperative targeted stretching ‘micro breaks’ (TSMBs) [21]. These are designed to interrupt extended periods of muscle loading and poor posture to prevent lactate build-up and fatigue. Although studies have demonstrated some improvement in mental focus and reduction in musculoskeletal pain and fatigue with TSMBs [21, 28], complete effectiveness in the long term is yet to be seen. In the case of RAS, an ergonomic operating chair may help improve comfort by maintaining a correct posture while operating [29].

### Emergence of robotic surgical systems

Robotic surgical systems have emerged as a feasible option to help overcome the mechanical limitations of conventional MAS instruments, offering improved visualisation, dexterity and precision [30]. Robotic systems have also negated some of the ergonomic challenges facing surgeons. Operating surgical instruments remotely, with the option to sit or stand, has eliminated the need to adopt static, hunched positioning when performing MAS. In the sitting position, an open-console design promotes the adoption of an upright posture and neutral pose minimizing musculoskeletal discomfort; the option to vary positioning is a key ergonomic advantage. However, systems with a closed-console design and uncomfortable controller hand grips leave room for ergonomic improvement [10]. Our finding that 90% of surgeons regularly performing RAS experience discomfort further supports that robotic systems should be more ergonomically designed. Future studies could explore differences in ergonomics between RAS and MAS from the perspective of surgeons performing both.

### Study strengths

A large international surgeon population representing multiple surgical specialties and techniques completed this survey, allowing fair representation of outcomes and robust comparisons. Survey questions were open-ended where possible to give surgeons the option to provide detailed, explanatory answers, including insights such as perceived age of peak performance and likelihood of early retirement, which are not widely reported in the literature.

### Study limitations

Given that the survey’s ten questions were designed to be completed in five minutes, it is difficult to fully assess causal factors and there is a risk of over-interpretation of the results. No formal psychometric validation analyses were carried out during the development of the survey, and the subjective nature of pain and discomfort can yield high variance in results. Although surgeon recruitment was carried out by an independent specialist, market research studies are associated with several inherent biases. For example, the wording of the survey’s questions may have been subject to confirmation bias, whereby the researcher is convinced of a hypothesis and responses are shaped to confirm this outcome [31]. Additionally, answers may be subject to sponsor bias, where exaggeration or scaling back of responses can result from respondents being aware of the study sponsor [32]. Finally, the surgeon population in this study did not include urologists, who frequently perform MAS. Perspectives from this surgical specialty would be important to consider in future research.

### Conclusion and future implications

This study demonstrates that ergonomic issues continue to persist in MAS and contributes to the evidence for the unmet needs of surgeons. Most surgeons in this large population across three countries experienced some degree of discomfort at least sometimes, most commonly in the back, neck, shoulders and feet. A considerable proportion of these surgeons felt it likely they would consider early retirement as a result, highlighting that surgeon longevity is a key issue with conventional MAS. Innovative solutions are needed to reduce the physical burden on surgeons and prevent potential economic and societal impacts on healthcare systems. Novel robotic surgical systems may help improve MAS ergonomics; however, no system has yet successfully provided a complete solution.

## Supplementary Information

Below is the link to the electronic supplementary material.

Supplementary file1 (DOCX 176 KB)

Supplementary file2 (JPG 111 KB)

## Data Availability

The authors confirm that the data supporting the findings of this study are available within the article and its supplementary materials.
