# Supplementary material for: The burden of performing minimal access surgery: ergonomics survey results from 462 surgeons across Germany, the UK and the USA
Source: J Robot Surg. 2022 Feb 2;16(6):1347–54. doi: 10.1007/s11701-021-01358-6 (PMC9606063; doi:10.1007/s11701-021-01358-6)
Supplement: Supplementary file 1 — Supplementary file1 (DOCX 176 KB) [file 11701_2021_1358_MOESM1_ESM.docx]

**The Burden of Performing Minimal Access Surgery: Ergonomics Survey Results from 462 Surgeons across Germany, the UK and the USA**

Jonathan Morton, FRCS,^1^ Grant D. Stewart, FRCS^1,2^

*^1^Cambridge University Hospitals NHS Foundation Trust, Cambridge, UK*

*^2^Department of Surgery, University of Cambridge, Cambridge, UK*

**Corresponding author:** Jonathan Morton ([jonathan.morton@addenbrookes.nhs.uk](mailto:jonathan.morton@addenbrookes.nhs.uk)); Department of Colorectal Surgery, Addenbrookes Hospital, Cambridge University Hospitals NHS Foundation Trust, Cambridge, UK

**SUPPLEMENTARY MATERIALS**

**Supplementary Table S1: Ergonomics survey questions**

| **Demographic questions** | |
| --- | --- |
| Q1 | How old are you? *(Free text)* |
| Q2 | How many years have you been practising as a surgeon? *(Free text)* |
| Q3 | Which gender do you identify with? *(Free text)* |
| Q4 | Which of the following, if any, best describes your current medical specialty? |
|  | Gynaecology / colorectal / general (performing hernia repair) |
| Q5 | Which of the following types of surgery do you routinely conduct? |
|  | Laparoscopic/MAS surgery / open surgery / robot-assisted surgery |
| Q6 | How tall are you? *(Free text)* |
| Q7 | What is your surgical glove size? *(Multiple choice)* |
|  | XS / S / M / L / XL |
| **Study questions** | |
| Q1 | How often do you experience muscular or back pain as a result of practicing surgery? *(Multiple choice)* |
|  | Never / rarely / sometimes / frequently / every time I operate |
| Q2 | How physically comfortable or uncomfortable are you during surgery? *(Multiple choice)* |
|  | Very comfortable / comfortable / neither comfortable nor uncomfortable / uncomfortable / very uncomfortable |
| Q3 | If you feel discomfort, where are you mostly affected? *(Multiple choice)* |
|  | Back / neck / shoulders / feet / legs / hands / knees / arms / hips / elbows |
| Q4 | Why do you think this happens? *(Free text)* |
| Q5 | How frequently do you experience physical discomfort during surgery? *(Multiple choice)* |
|  | Never / rarely / sometimes / frequently / every time I operate |
| Q6 | Do you have any issues using your hands? *(Y / N)* |
| Q7 | What issues do you have using your hands? *(Free text)* |
| Q8 | How likely do you think it is you may need to retire early due to the physical impact on your own health of operating? *(Multiple choice)* |
|  | Not at all likely / not very likely / don’t know / fairly likely / very likely |
| Q9 | At what stage in your opinion, do surgeons reach their peak professional performance? *(Multiple choice)* |
|  | 30–35 / 35–40 / 40–45 / 45–50 / 50–55 / 55–60 / prefer not to say |
| Q10 | Have you ever personally had to consult a healthcare professional due to musculoskeletal injuries associated with minimal access surgery? *(Multiple choice)* |
|  | Yes / no / prefer not to say |

**Supplementary Table S2: Direct quotes for reasons for discomfort while performing MAS**

| Why do you think you feel discomfort during surgery? |
| --- |
| ‘Prolonged standing lasting several hours and working without a break’ |
| ‘Standing without much movement for prolonged periods of time’ |
| ‘Especially in laparoscopic procedures, a longer-term oblique posture (e.g., when standing sideways-on to the patient) occasionally results in neck tension pains’ |
| ‘Length of surgery as standing for a long time. My shoulders hurt when I do long laparoscopic surgeries’ |
| ‘Twisting my body into uncomfortable positions’ |
| ‘Standing for a long time in concentrated, forced posture under constant tension’ |
| ‘Unfavourable position/posture at the table’ |
| ‘Prolonged standing with a strained arm position and uneven weight distribution’ |
| ‘Positioning during laparoscopic surgery can cause difficult back positioning in gynaecology [procedures]’ |
| ‘Gynaecology surgery almost always results in poor ergonomic positioning, especially vaginal surgery. Repetitive movements are tiring and straining’ |
| ‘Due to the prolonged standing the body gets tired and you’d often be standing in an unfavourable position, as not all equipment is designed to fit tall surgeons’ |
| ‘Performing surgery mandates certain positions for longer periods of time, and certain positions that are unnatural. With a ‘vulnerable’ neck/back, those factors significantly increase discomfort/pain’ |
| ‘Due to the long, relatively static standing with minimal opportunity to move around’ |
| ‘Occasional pain due to the back being in a twisted position’ |
| ‘Tensing up over long periods, especially when operating laparoscopically, uncomfortable posture’ |
| ‘In particular during laparoscopic procedures, a crooked posture (e.g., when lateral to the patient) leads to long-term muscle tensions and pain in the neck area’ |
| ‘Prolonged standing in one position, in particular during endoscopic procedures’ |
| ‘I find myself super-extending my knees while I operate without even realising and, after standing for 2–3 hours, it becomes really painful’ |
| ‘I am 6ft 2” and the table is often too low for my height, so I have to bend my neck down far’ |
| ‘I especially feel neck and shoulder discomfort during direct view (without a camera) laparoscopy. Sometimes I can see better that way, but it is a very awkward position. Otherwise, I mostly feel discomfort during long procedures’ |
| ‘Positioning of patients and instruments are not necessarily ergonomically designed, nor designed with female surgeons in mind’ |
| ‘Shoulder and neck positions for vaginal hysterectomy, especially with an assistant, is challenging. Also shoulders from reaching to use laparoscopic ports. Although I do not use a microscope frequently, when I do it can cause neck pain if not meticulously positioned’ |
| What issues do you have using your hands? |
| ‘After back-to-back surgeries my thumb and index finger get very swollen and lose their complete range of motion’ |
| ‘Bilateral carpal tunnel syndrome’ |
| ‘Joint issues’ |
| ‘Occasional pain and stiffness’ |
| ‘I get some pain at sites of tendons in my wrist’ |

**Supplementary Figure S1: Frequency of discomfort by surgeon height**


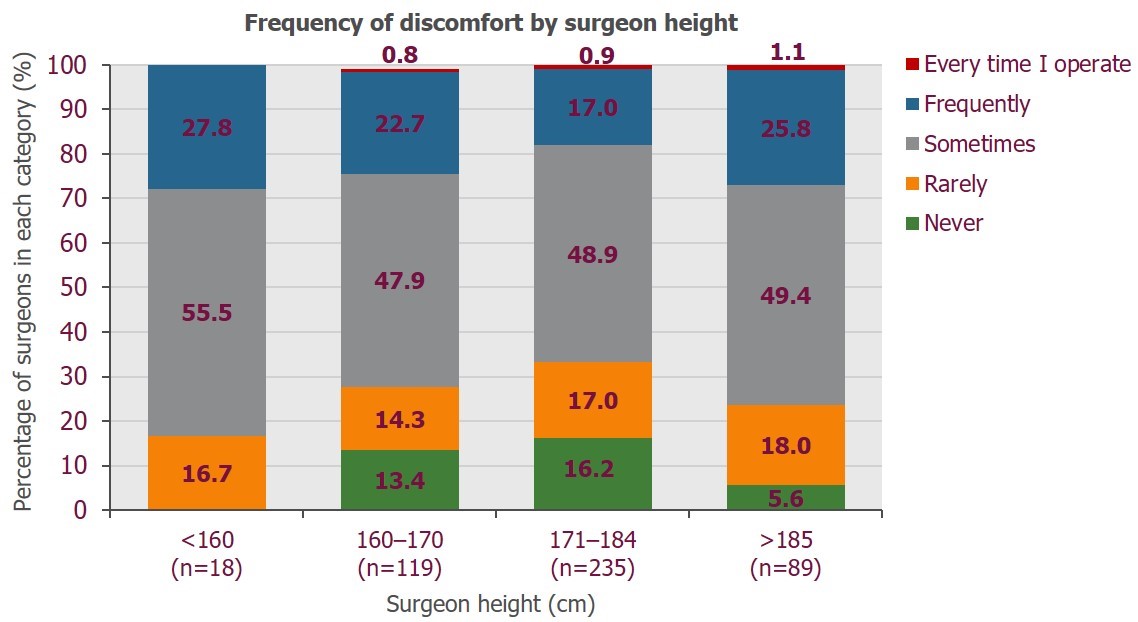


Frequency of discomfort while performing surgery stratified by surgeon height category.
