# Supplementary figures and images for: The burden of performing minimal access surgery: ergonomics survey results from 462 surgeons across Germany, the UK and the USA
Source: J Robot Surg. 2022 Feb 2;16(6):1347–54. doi: 10.1007/s11701-021-01358-6 (PMC9606063; doi:10.1007/s11701-021-01358-6)

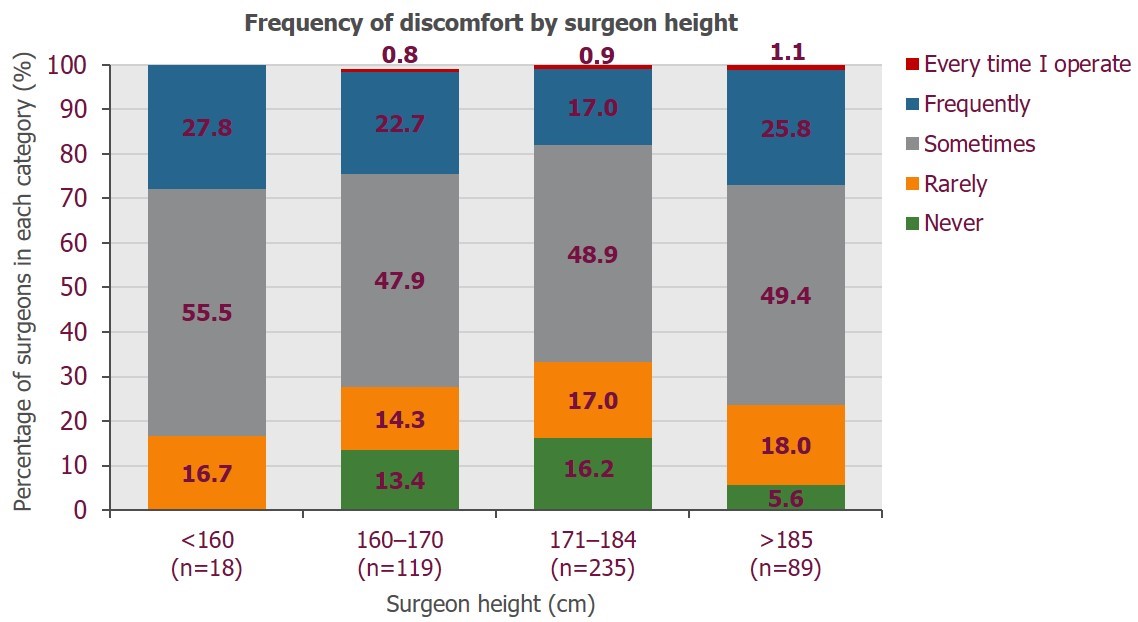

Supplement: Supplementary file 2 — Supplementary file2 (JPG 111 KB) [file 11701_2021_1358_MOESM2_ESM.jpg]
